# Microcephaly genes evolved adaptively throughout the evolution of eutherian mammals

**DOI:** 10.1186/1471-2148-14-120

**Published:** 2014-06-05

**Authors:** Stephen H Montgomery, Nicholas I Mundy

**Affiliations:** 1 Department Genetics, Evolution & Environment, University College London, Gower Street, London WC1E 6BT, UK; 2 Department Zoology, University of Cambridge, Downing Street, Cambridge CB2 3EJ, UK

**Keywords:** *ASPM*, Adaptive evolution, Brain size, *CDK5RAP2*, Mammals, Microcephaly genes, Neurogenesis

## Abstract

**Background:**

Genes associated with the neurodevelopmental disorder microcephaly display a strong signature of adaptive evolution in primates. Comparative data suggest a link between selection on some of these loci and the evolution of primate brain size. Whether or not either positive selection or this phenotypic association are unique to primates is unclear, but recent studies in cetaceans suggest at least two microcephaly genes evolved adaptively in other large brained mammalian clades.

**Results:**

Here we analyse the evolution of seven microcephaly loci, including three recently identified loci, across 33 eutherian mammals. We find extensive evidence for positive selection having acted on the majority of these loci not just in primates but also across non-primate mammals. Furthermore, the patterns of selection in major mammalian clades are not significantly different. Using phylogenetically corrected comparative analyses, we find that the evolution of two microcephaly loci, *ASPM* and *CDK5RAP2*, are correlated with neonatal brain size in Glires and Euungulata, the two most densely sampled non-primate clades.

**Conclusions:**

Together with previous results, this suggests that *ASPM* and *CDK5RAP2* may have had a consistent role in the evolution of brain size in mammals. Nevertheless, several limitations of currently available data and gene-phenotype tests are discussed, including sparse sampling across large evolutionary distances, averaging gene-wide rates of evolution, potential phenotypic variation and evolutionary reversals. We discuss the implications of our results for studies of the genetic basis of brain evolution, and explicit tests of gene-phenotype hypotheses.

## Background

For over a decade researchers interested in the genetic basis of brain evolution have sought clues in the molecular evolution of genes associated with the neurodevelopmental disorder, primary microcephaly [1-8]. Microcephaly is a congenital disorder characterized by an early cessation of brain growth, specifically affecting cortical development. Mendelian inheritance of microcephaly has now been linked to deleterious mutations at seven unlinked loci [8-15]. These loci encode proteins with central roles in neurogenesis, largely in the formation and function of the centrioles, which in turn control the way in which neural progenitor cells divide [16-18]. Disruption of these crucial functions causes microcephaly, and it is hypothesized that modification of their function through evolutionary time could tip the cell fate switch towards greater neurogenic output, underpinning the evolution of larger brains.

Much of the early focus on the molecular evolution of microcephaly genes centered on the role of two loci (*ASPM* and *MCPH1*) in human evolution [19,20]. Early studies suggested an increase in the rate at which these genes evolved along the lineage leading to humans [1]. Intriguingly, as more microcephaly loci were identified each has been shown to evolve adaptively [5,6] suggesting they may be a persistent target of positive selection. In addition, as more species were incorporated into analyses the signature of positive selection extended beyond humans first to great apes [4], then to all anthropoid primates [7,8]. Similarly, the potential phenotypic relevance of this selection was extended from a role in the rapid expansion of human brain size [1-3] to a more widespread, conserved role in primate brain evolution [7,8]. This shift has been accompanied by more rigorous hypothesis testing on larger datasets [7,8,21]. For example, it has previously been found that the molecular evolution of *ASPM* and *CDK5RAP2* co-evolves with brain mass, particularly neonatal brain mass [8]. The association between *ASPM* evolution and brain mass is particularly interesting as it is found in primate clades which experienced both increases and decreases in brain mass [8,21]. Elsewhere, tentative evidence has been found linking the evolution of *MCPH1* to sexual dimorphism in brain mass in primates [22], a surprising finding supported, in part, by human population studies of sex-specific associations between SNPs in microcephaly genes and brain size [23,24] and functional analyses of base pair substitutions that may interact with sex-specific developmental pathways [25].

Other studies have extended the taxonomic scope beyond primates to test the hypothesis that microcephaly genes may contribute to the evolution of brain size in other mammals. Notably, so far these studies have been limited to species with relatively large brains. In cetaceans, both *MCPH1* and *ASPM* have been shown to have evolved under positive selection [26,27]. However, evidence linking selection on either locus to brain size in cetaceans is lacking [26,28]. In addition, exclusively studying large brained clades inevitably leads to an ascertainment bias. Indeed, there is evidence that both of these genes evolved under positive selection across placental mammals [26,28]. If this is the case, there are clear implications for our understanding of brain evolution. First, if there is an evolutionary link between microcephaly loci and changes in neurogenesis, such pervasive selection may suggest a conserved genetic basis to some aspects of mammalian brain size evolution. Second, the diversity of mammalian brain sizes could provide a good comparative framework in which to test for gene-phenotype co-evolution. Finally, if microcephaly genes do have a conserved evolutionary role in brain size, or any other phenotype, the results of functional assays within and between more practically tractable species than primates, such as rodents, may generalize to other mammals.

In this study, we examine patterns of molecular evolution in seven microcephaly genes across placental mammals, including the first comprehensive interspecific analysis of three of these loci, *STIL*, *CEP152* and *WDR62,* and the first mammal-wide analysis of two further loci, *CDK5RAP2* and *CENPJ*. We perform tests for adaptive evolution within primates, across non-primate mammals and across the combined data to test how pervasive the signature of positive selection is. We further test whether the pattern of evolution of these genes differ in primates compared to non-primates, and finally provide a preliminary assessment of the link between microcephaly genes and brain size in non-primate clades. We find a signature of positive selection that is strong and widespread for all but one of the loci and identify intriguing evidence of an association between two loci and neonatal brain mass in two non-primate clades. Greater sampling both at the molecular and phenotypic level are necessary to perform robust tests of gene-phenotype associations to confirm this hypothesis, but this preliminary extension of the role of microcephaly genes in brain evolution beyond primates may have a number of implications.

## Results

### Tests for positive selection

**Figure 1:**
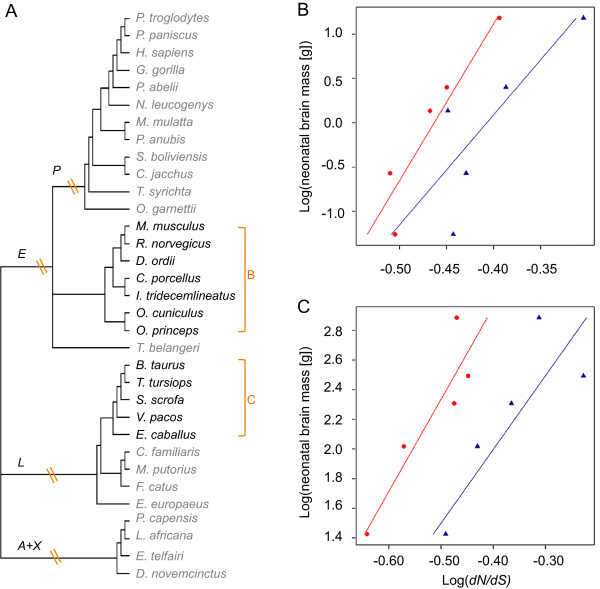
**Species included in tests of adaptive evolution and gene-phenotype associations. A)** Unrooted phylogeny from Meredith *et al*. (2011) used in the PAML analyses. P = Primates, E = Euarchontoglires, L = Laurasiatheria, X + A = Xenarthra + Afrotheria. The bracketed clades refer to Glires (B) and Euungulata (C). Panels **B)** and **C)** show the relationship between root-to-tip *dN/dS* and neonatal brain sizeg in Glires and Euungulata respectively, for the two genes with the most consistent pattern across the two groups, *ASPM* (red circles) and *CDK5RAP2* (blue triangles). Regression lines are derived from the PGLS analysis and account for phylogenetic non-independence between the datapoints.

Full coding sequences for seven loci (*ASPM*, *CDK5RAP2*, *CENPJ*, *CEP152*, *MCPH1*, *STIL* and *WDR62*) were obtained for 12 primates and 21 non-primate eutherian mammals (Figure 1a). Two site model tests were applied to these datasets, the M1a/M2a and M8a/M8 pairs implemented in PAML [29]. These allow the ω (estimate of *dN/dS*) to vary among sites but not across lineages [30,31].

#### Across primates

Across the 12 primate species for which full coding sequences are available for all 7 loci, four loci - *ASPM*, *CDK5RAP2*, *CENPJ* and *CEP152* - show a consistent signature of positive selection (Table 1a). Of the three that do not, one of these, *MCPH1*, has previously been shown to have evolved under positive selection across a larger dataset of partial coding sequence in anthropoid primates [8] suggesting that the lack of significance may be due to the small sample size. We therefore expanded the anthropoid dataset to include 18 species by amplifying selected exons of the remaining two loci (*STIL, WDR62*), targeting regions with high *dN/dS* based on a sliding window analysis of the full coding sequence (Additional file 1: Figure S1). Consistent with an effect of sample size these additional data yield evidence for positive selection acting on *WDR62* (M1a/M2a Likelihood ratio (LR) = 11.921, p = 0.003; M8a/M8 LR = 11.903, p = 0.001). The results for *STIL* are inconsistent between tests (M1a/M2a LR = 3.940, p = 0.139; M8a/M8 LR = 4.688, p = 0.030).

**Table 1:** Site model tests for positive selection

**A) Primates (n = 12)**										
	**Likelihood ratio**		**p-value**		**M2a**		**M8**		**Corrected p-value**	
**Locus**	**M1a/M2a**	**M8/M8a**	**M1a/M2a**	**M8/M8a**	**prop >1**	**dN/dS >1**	**prop >1**	**dN/dS >1**	**M1a/M2a**	**M8/M8a**
*ASPM*	14.015	11.390	0.001	0.001	0.007	5.351	0.020	3.504	0.005	0.005
*CDK5RAP2*	36.471	36.381	<0.001	<0.001	0.069	3.092	0.093	2.845	<0.001	<0.001
*MCPH1*	4.355	4.815	0.113	0.028	0.018	3.533	0.033	2.931		
*CENPJ*	23.603	24.768	<0.001	<0.001	0.107	2.399	0.144	2.242	<0.001	<0.001
*STIL*	3.041	3.159	0.219	0.075	0.008	4.828	0.015	3.775		
*CEP152*	9.359	9.303	0.009	0.002	0.113	1.815	0.194	1.576	0.036	0.008
*WDR62*	3.424	3.590	0.180	0.058	0.136	1.307	0.134	1.319		
**B) Non-primate mammals (n = 21)**										
	**Likelihood ratio**		**p-value**		**M2a**		**M8**		**Corrected p-value**	
**Locus**	**M1a/M2a**	**M8/M8a**	**M1a/M2a**	**M8/M8a**	**prop >1**	**dN/dS >1**	**prop >1**	**dN/dS >1**	**M1a/M2a**	**M8/M8a**
*ASPM*	48.679	59.244	<0.001	<0.001	0.011	2.802	0.036	1.798	<0.001	<0.001
*CDK5RAP2*	50.860	50.261	<0.001	<0.001	0.029	2.384	0.104	1.578	<0.001	<0.001
*MCPH1*	66.740	62.251	<0.001	<0.001	0.067	2.230	0.101	1.691	<0.001	<0.001
*CENPJ*	22.289	26.464	<0.001	<0.001	0.016	2.660	0.058	1.623	<0.001	<0.001
*STIL*	0.000	2.274	1.000	0.132	0.094	1.000	0.023	1.508		
*CEP152*	48.651	39.601	<0.001	<0.001	0.020	2.447	0.098	1.467	<0.001	<0.001
*WDR62*	0.000	13.425	1.000	<0.001	0.228	1.000	0.010	2.148		<0.001
**C) All mammals (n = 33)**										
	**Likelihood ratio**		**p-value**		**M2a**		**M8**		**Corrected p-value**	
**Locus**	**M1a/M2a**	**M8/M8a**	**M1a/M2a**	**M8/M8a**	**prop >1**	**dN/dS >1**	**prop >1**	**dN/dS >1**	**M1a/M2a**	**M8/M8a**
*ASPM*	87.071	86.279	0.000	0.000	0.015	2.662	0.046	1.705	0.000	0.000
*CDK5RAP2*	101.844	86.044	0.000	0.000	0.035	2.352	0.115	1.597	0.000	0.000
*MCPH1*	94.456	76.822	0.000	0.000	0.077	2.145	0.107	1.631	0.000	0.000
*CENPJ*	40.527	44.551	0.000	0.000	0.027	2.383	0.081	1.563	0.000	0.000
*STIL*	0.000	8.888	1.000	0.003	0.000	167.895	0.032	1.553		0.021
*CEP152*	97.383	70.324	0.000	0.000	0.022	2.604	0.062	1.698	0.000	0.000
*WDR62*	0.000	13.163	1.000	0.000	0.000	41.266	0.009	2.011		0.000

#### Across mammals

Consistent evidence for positive selection is found across non-primate mammals under both site model tests for 5 loci - *ASPM*, *CDK5RAP2*, *MCPH1*, *CENPJ* and *CEP152* (Table 1b). When the site model tests are repeated after incorporating the 12 primate sequences extensive positive selection is again found, with the M8a/M8 test for *STIL* also becoming narrowly significant (Table 1c). Among the loci which experienced positive selection across mammals the proportion of sites targeted by selection varies from ~2% to ~10% with *dN/dS* estimates for these sites typically between 1.5 and 2.5.

### Diversifying selection on microcephaly loci across mammals

We next examined patterns of divergent selection in different mammalian clades using branch and clade models. Branch models allow *dN/dS* to vary across branches in the phylogeny but not across sites [32,33], whereas clade models allow a proportion of sites to undergo divergent selection pressures in two or more clades defined *a priori*[34]. Comparisons of the gene-wide average *dN/dS* between primates and non-primate mammals are significant for all microcephaly loci except *WDR62* (Additional file 2: Table S2a)*.* Whilst this may suggest a greater influence of positive selection in primates, the clade models do not support this general conclusion. Here, only two loci, *CDK5RAP2* and *WDR62*, are supported as having a proportion of sites under significantly different selective regimes between primates and other mammals (Additional file 2: Table S2b). It therefore seems likely that the significant branch model results reflect technical issues, such as branch length, or neutral effects such as differences in population size or life history [35-37]. The possibility of divergent selective pressure was next explored on a wider taxonomic scale comparing three major eutherian clades: the Euarchontoglires (n = 20), the Laurasiatheria (n = 9), and the Afrotheria + Xenarthra (n = 4) (see Figure 1a for these clades). Under the branch models there is little evidence for divergent selective regimes between these three clades and clade model tests are only significant for two loci, *ASPM* and *MCPH1* (Additional file 2: Table S2c). The overarching pattern of selection on microcephaly loci is therefore a consistent signature of positive selection across all eutherian mammals, perhaps with the exception of *STIL*, but with relatively little evidence of clade-specific differences in overall rates of evolution.

### Linking molecular and phenotypic evolution

Understanding the phenotypic relevance of this widespread positive selection is clearly of major interest. Given the long held hypothesis that selection on microcephaly genes in primates is linked to brain expansion [1] and the comparative evidence linking rates of evolution of *ASPM* and *CDK5RAP2* to variation in brain mass across anthropoid primates [8,21], a particular interest is the possibility that microcephaly genes may have a conserved role in mammalian brain evolution [26,27,38]. One approach to test a link between molecular evolution of candidate genes and brain size has been to compare pairs of species that differ in brain size (e.g. [39]). In our phylogeny three episodes of brain expansion are relatively well documented in the fossil record and form taxon-pairs with smaller brain species; the expansion of human [40,41], dolphin [42,43] and elephant brain sizes [44]. Branch models were used to test for significant differences in *dN/dS* between the bottlenose dolphin and the cow (divergence c. 50 my), between the elephant and the hyrax (divergence c. 80 mya), and between humans and the bushbaby (chosen to provide a similar branch length, divergence c. 80 mya). Only two loci showed significant differences between elephant and hyrax (Additional file 2: Table S3a), of which only one, *MCPH1*, had a higher *dN/dS* in the elephant lineage. Comparing the dolphin and cow branches no loci showed significant differences, though *CDK5RAP2* approached significance and had a higher *dN/dS* on the dolphin branch. Finally, only *CEP152* showed a significantly higher *dN/dS* along the human lineage compared to the bushbaby lineage. Hence, there is no consistent pattern of acceleration of microcephaly evolution along lineages leading to selected large brain species, compared to their smaller brained sister-lineage as represented in this dataset. Again, as these branches are relatively long *dN/dS* estimates are likely influenced by variation in life history and population size.

An alternative test of a gene-phenotype link is to take a more quantitative approach and test for co-evolution between molecular rates and the trait of interest. We tested for associations between root-to-tip *dN/dS* and two traits: neonatal and adult brain mass. Comparisons between any associations with these traits are informative as we expect genes involved in the evolution of neurogenesis to show a stronger relationship with neonatal brain mass as mammalian neurogenesis is predominantly prenatal [45-47]. We applied this test to three sub-clades within the mammalian phylogeny; anthropoid primates (n = 8), the focus of previous studies, and two additional grand-orders [48], the Glires (Rodentia + Lagomorpha, n = 5) and Euungulata (Perissodactyla + Cetartiodactyla, n = 5) chosen to reflect a trade-off between a relative consistency in life history parameters, the number of species and sample density. Within primates we found no evidence for an association with any of the seven loci (Additional file 2: Table S3b). Using larger datasets of partial coding sequence the evolution of *ASPM* and *CDK5RAP2* have been linked to brain size [8,21]. We repeated the tests using only the exons sequenced for previous studies [8] and again found no association suggesting sample size, which was much lower in the current study, and the phenotypic diversity within the dataset contribute to the difference in results between the present analysis and previous studies.

Within the Glires and Euungulata datasets there is an intriguing pattern suggesting a link between brain size and selection on some microcephaly loci. Although based on small samples sizes, these results provide the first evidence of a microcephaly gene-phenotype association outside primates. In Glires only *ASPM* is significantly associated with neonatal brain mass (t_3_ = 2.624, p = 0.039), whilst *CDK5RAP2* (t_3_ = 2.235, p = 0.056) and *STIL* (t_3_ = 2.192, p = 0.058) show non-significant trends. In all three cases the strength of the association is reduced, or lost, with adult brain mass (Additional file 2: Table S3). Within Euungulata *ASPM* (t_3_ = 3.639, p = 0.018), *CDK5RAP2* (t_3_ = 2.859, p = 0.032) and *WDR62* (t_3_ = 2.824, p = 0.033) show significant associations with neonatal brain size and again the significance falls when adult brain size is considered (Additional file 2: Table S3b). In contrast *STIL* shows a significant association with adult brain size (t_3_ = 3.785 p = 0.016), which is reduced to a non-significant trend when neonatal brain size is considered (t_3_ = 1.911, p = 0.076).

## Discussion

Our results indicate that the majority of loci linked to microcephaly, a severe neurodevelopmental disorder, have been targeted by positive selection throughout the evolution of eutherian mammals, in both primates and non-primates. Given the large evolutionary time under consideration it is remarkable that such a consistent pattern should be found on a functionally related set of genes that share a key role in neural development. Only *STIL* shows a weak or inconsistent signature of adaptive evolution. Given the paucity of brain expressed coding-genes with high rates of evolution identified in the majority of genome scans (e.g. [49-52] but see [53]) this raises the intriguing possibility that microcephaly genes are hotspots for positive selection among brain expressed coding genes. Whether this is true or not will require a further examination of genome wide patterns of selection across a greater number of species, as no study has included comparable numbers of species. A recent exome-wide analysis across 7 species of primates, to our knowledge the largest to date, did not report any enrichment for brain-expressed genes or neurodevelopmental processes among positively selected genes [54], nor did a study of six mammalian genomes [52]. However, these sample sizes are towards the lower limit at which site-based models have power to detect positive selection [55]. To fully assess whether microcephaly genes are targeted by positive selection more frequently than other neurodevelopmental genes it will be necessary to perform genome-wide analyses of the selective regimes acting on mammalian protein coding genes with much larger sample sizes than previous studies.

The frequent targeting of microcephaly genes by selection also raises important questions about the functional effects of substitutions in these loci. The majority of the microcephaly genes contribute to the development and function of the spindle poles, or astral microtubule network [56-58], and disruption of this function is linked to changes in spindle or microtubule behavior [59,60]. The spindle poles play a key role in the cell fate switch of neural progenitor cells. In the developing brain a pool of neural progenitor cells undergo successive symmetric and proliferative divisions, their number increasingly exponentially [61]. After a certain number of divisions these cells begin to divide asymmetrically, with each division contributing a neuron to a radial column of cortical neurons before terminally dividing into two neurons [61,62]. This switch between proliferative, symmetric divisions to asymmetric neurogenic divisions is controlled by the angle of cell division, which is in turn controlled by the spindle poles [59,61]. Hence, functional changes in microcephaly genes could conceivably alter the duration of symmetric divisions to ultimately change the number of neurons produced during brain development.

A key role for modification of this cell fate switch is consistent with evo-devo models of brain expansion [45,62-64]. The Radial Unit Hypothesis suggests a general mechanism for rapidly increasing brain size in mammals is to prolong the period of symmetric divisions, resulting in more radial units of neurons and an expanded volume [45,63]. Microcephaly genes have precisely the functions the Radial Unit Hypothesis would predict as being targeted during episodes of brain expansion. Notably, another candidate gene, *NIN*, which functions in the maintenance of asymmetric divisions of neural progenitor cells [65], and has not been linked to microcephaly, also shows a signature of adaptive molecular evolution across anthropoid primates [66]. Maintaining this asymmetric cell division would result in larger numbers of neurons/radial unit and comparative data suggest an association between selection at this locus and interspecific variation in the number of neurons/unit area in the cortex, a suggested proxy for the number of neurons per radial unit [66]. Both cell fate switches highlighted by the Radial Unit Hypothesis therefore involve proteins that were targeted by positive selection across long periods of evolutionary time.

Although there is some variation in cell developmental pathways leading to neuron production among mammals, such as the emergence of additional progenitor cells associated with increased gyrification [67], much of the developmental program is conserved [61,62,68,69]. Comparative data across mammals also suggest the timing of brain development is conserved [70] indicating strong constraints act on brain development, limiting the potential ways in which selection can modify brain size and structure. These constraints may be due to pleiotropic effects of shared developmental pathways or to adaptive, functional co-evolution [70,71] but regardless, provide little reason to suspect genes targeted by selection in relation to primate brain evolution should differ from those targeted in non-primate mammals. Such convergence, or parallelism, in the genetic basis of mammalian phenotypes may be more widespread than perhaps expected, with examples including sensory perception [72], energy metabolism [39], digestive enzymes [73], immunity genes [74] and coloration [75].

The results of our phylogenetic comparative analyses provide direct evidence that evolution of brain size is indeed linked to four microcephaly genes (*ASPM*, *CDKRAP2, STIL, WDR62*) in two mammalian clades, Glires and Euungulata. Furthermore, as predicted by the neurodevelopmental models above, the relationship is generally stronger for neonatal brain size, a time point by which most neurons have already arisen [46,47], than adult brain size. These results raise the possibility that *ASPM* and *CDK5RAP2* play a consistent role in mammalian brain evolution, as these two loci have previously been implicated in brain evolution in primates [8,21] (the failure to obtain a positive relationship for these two genes in primates here is most likely related to the sample size).

However, we stress that the sample sizes are small, the significance of our gene-phenotype tests are all >0.01, and it will be necessary to confirm and further explore these results with larger datasets. Regardless, the key implication of our analysis is that diversifying evolutionary studies of brain size beyond enigmatic, large brained clades may offer new avenues for testing evolutionary or functional hypotheses. It is likely however, that testing such gene-phenotype hypotheses over large evolutionary distances will be a challenging endeavor for a number of reasons [28]. First, effects of non-adaptive processes such as variation in life history or population size may affect *dN/dS*[35-37,76] introducing noise to any genuine gene-phenotype association making larger, more densely sampled datasets desirable. Similarly, variation in phenotypic structure, such as neuron density, which may affect the linearity of the relationship between brain mass and neuron number [77,78], may impact upon gene-phenotype comparisons across large evolutionary distances. Careful consideration is therefore needed as to what phenotype is most relevant, and molecular studies should target species where phenotypic data is available, when these data are a limiting factor.

Second, evolutionary reversals may be common in some mammalian clades and this may obscure gene-phenotype associations. For example, if a gene is targeted by selection during both increases and decreases in brain size there could be a mismatch between high rates of evolution at the molecular level and a small perceived difference in brain size. Understanding the evolutionary history of a phenotype then becomes a key component of the search for that phenotype’s molecular basis. An example of this comes from callitrichids, a subfamily of New World Monkeys that experienced a decrease in brain mass [41] and gyrencephaly [79,80] in association with major episodes of phyletic dwarfism [81,82]. Callitrichids appear as outliers to the positive association between brain mass and *dN/dS* for *ASPM* across anthropoids [8], but when considered alone show a negative association between brain mass and *dN/dS* for *ASPM* suggesting functional changes in *ASPM* may have contributed to decreases in brain mass in this clade [21]. This is also a potential explanation for the lack of an evolutionary association between *ASPM* and brain mass in cetaceans, where reversals are more common [26-28]. A third problem could be encountered if a gene is associated with a phenotype but has an intermittent role in its evolution. A potential example here is *CDK5RAP2* that coevolves with brain mass in primate taxa where it has increased [8], but not when it has decreased [21]. In the present case the positive trends found in Glires and Euungulata would be expected if phenotypic differences within these clades are mostly due to increases in brain size on lineages leading to larger brained species, rather than decreases leading to smaller brained species.

Finally, a major challenge may occur when the gene-phenotype association is limited to a small subset of domains or sites within a gene. Site-based methods for detecting positive selection were developed because it is thought that selection is unlikely to act across a whole gene equally [30,31]. This positive selection is presumably associated with some phenotypic, functional or fitness-related change, and it is therefore likely that gene-phenotype associations may be limited to a subset of domains or codons. Developing methods, which are capable of testing gene-phenotype associations on a site-by-site basis, or which incorporate a form of sliding window analysis, may be a worthwhile endeavor (e.g. [83]). Again, to gain sufficient statistical power such analyses will require large, densely sampled datasets.

The pervasive signature of positive selection across mammals, combined with the limited evidence of divergence in selection pressures, suggests results from experimentally tractable clades may be applicable to wider taxonomic groups. The link between selection on microcephaly genes and the evolution of brain size has yet to be confirmed (or rejected) by functional data. Mice transgenic for *ASPM*, and in vitro assays for *MCPH1* confirm changes in the coding sequence have functional affects [25,84]. However, although the human sequence of *ASPM* rescued the phenotype of transgenic mice with a disrupted copy of *ASPM* it did not lead to an increase brain size [84]. This has been interpreted as being indicative of functional conservation [84], but without the reciprocal experiments this conclusion may be premature. It could be, for example, that the effects of microcephaly genes are background dependent, or are combinatorial such that changing individual genes has only a minor or no affect in isolation. Given the importance placed in functional confirmation of gene-phenotype associations a shift towards examining phenotypic diversity within experimentally tractable clades may be worthwhile. For example, greater sampling density in rodents could permit comparative analyses to test for positive selection, and subsequently test for gene-phenotype associations. A range of data exists for a number of rodents that vary widely in brain size, neuron number and gyrencephaly [77,85], and the results presented above for Glires provide some encouragement for pursuing studies in this clade. Both micro and macroevolutionary gene-phenotype associations could be performed and, if an association were found, supporting evidence could be sought through evolutionary developmental studies. Rather than doing one-way human/mouse transgenic experiments [84], it may be just as informative to perform, for example, reciprocal rat/mouse transgenic experiments. Although we note that recent developments in *in vitro* human organoids may render two-way human/mouse transgenics technically feasible [86], ethical considerations may still limit this approach. Regardless of the results of such a study, greater sampling within clades with smaller brains will provide a useful comparison with larger brained mammalian orders, such as primates and cetaceans, and are clearly necessary to avoid ascertainment biases.

## Conclusions

We have shown that microcephaly genes have experienced pervasive positive selection not just across primates but across placental mammals. We find little evidence to suggest that these loci experienced divergent selective pressures in different clades, which may suggest conservation in function and imply a common phenotypic relevance for this wide spread positive selection. Developmental models of cortical expansion and evidence for conservation in brain developmental pathways provide a clear basis with which to hypothesize that the phenotype of relevance is brain mass, or more specifically the number of neurons produced during cortical neurogenesis. We provide evidence to support this hypothesis in Glires and Euungulata that, combined with previous work in primates, suggests that this phenotypic association may be common across mammals. Whilst several challenges face attempts to test gene-phenotype hypotheses, the persistent signal of positive selection should permit useful studies in more experimentally tractable species.

## Methods

### Data, alignment and phylogeny

Full coding sequence for mammalian species were collected from Ensembl and GenBank. Only species that were available for all seven loci (*ASPM*, *CDK5RAP2*, *CENPJ*, *CEP152*, *MCPH1*, *STIL* and *WDR62*) were included to permit fair comparisons between loci. Ensembl/Genbank accession IDs for these species are shown in Table S1. In total we obtained full coding sequence for 12 primates and 21 non-primate eutherian mammals. No marsupials were included due to a lack of species with data for all seven loci.

Additional sequence data were generated to test if increased sampling results in stronger evidence for positive selection for two loci in primates. In these cases, regions for amplification were chosen based on peaks of high *dN/dS* identified using a sliding window analysis performed across the alignment of the full coding sequence from anthropoids using SWAAP [87], the Nei and Gojobori [88] method with a window size of 150 codons and a step size of 15 codons. From this alignment primers were designed in conserved regions using Primer3Plus [89]. Genomic DNA samples were extracted from tissue samples using DNeasy kits (QIAGEN UK, Crawley, UK) for a previous study by the same authors [88]. Ethical approval was not required as all DNA was obtained from archived tissue samples taken from animals that were captive born in the UK and that died of natural causes or that were euthanized for reasons unrelated to the current research. Tissue samples were originally obtained from Andrew Kitchener at the National Museums of Scotland, or Leona Chemnick at the Center for Reproduction of Endangered Species, San Diego Zoo, with permission to use the samples in molecular biology studies.

Polymerase chain reactions (PCR) were performed using standard protocols and BIOTAQ DNA polymerase PCR kits (BIOLINE, London, UK). PCR products were purified using Qiagen QIAquick PCR purification kits. Cycle sequencing on both strands was carried out using BIG DYE v. 3.1 (PE Biosystems) under standard conditions. Precipitated DNA was sent to the Oxford Sequencing Centre (Dept. of Zoology, University of Oxford) for sequencing runs. In total we amplified 5 exons (7, 13, 15, 17 and 18) from *STIL*, totaling c.2400 bp and one 520 bp exon (30) from *WDR62* from an additional 8 species which were added to the 10 available. The site model tests for positive selection were repeated using an alignment of these 18 anthropoids. Sequences were aligned using MUSCLE in MEGA 5.0 [90]. All alignments were filtered to remove poorly aligned sequence, removing short stretches of sequence surrounded by gaps and regions with an excessive number of substitutions in a short sequence. All alignments are available by request from SHM. For the molecular evolution analyses the mammalian phylogeny was taken from two published mammalian phylogenies, which produced comparable results [91,92].

### Molecular evolution analyses

A common measure used to infer selection pressures acting on coding regions of genes is the ratio of rates of non-synonymous to synonymous fixed base changes. Estimation of *dN/dS* ratios (ω) was carried out using a codon-based maximum likelihood method (codeml in PAML version 4.7 [29]). Several analyses were performed to test the hypothesis that the candidate loci have experienced positive selection or that rates of evolution vary between clades. Nested models were tested by comparing the likelihood ratio statistic (−*2(Log[Lh(null model)]–Log[Lh(alternative model)]*) to critical values of the Chi-square distribution using degrees of freedom as the difference in the number of parameters estimated by each model. For each test we correct for multiple testing using the sequential Bonferroni method, with n = 7, the number of loci tested.

#### Tests for positive selection

To detect positive selection we implemented the site models. These allow the ω to vary among sites but not across lineages [30,31]. The site model tests for positive selection can be carried out using two pairs of models. The first pair compare Model M1a and Model M2a [55,93]. Model M1a (NearlyNeutral) allows sites to fall into two categories with ω <1 (purifying selection) and ω = 1 (neutral evolution), whilst model M2a (PositiveSelection) allows sites to fall into three categories with ω <1, ω = 1 and ω >1 (positive selection) [55]. The second pair compares Model 8a and Model 8 [93,94]. These models use the beta distribution to describe the numbers of sites across different categories of ω. M8 has 11 site classes (10 from the beta distribution plus 1 additional class), where one of these classes may have an ω >1. In Model 8a this latter class is restricted to have an ω equal to 1. The critical Likelihood Ratio boundaries of significance for this test are 2.71 at 5% and 5.41 at 1%, but here we calculate significance using a chi squared test with a more conservative one degree of freedom [93]. The M1a-M2a test has slightly lower false positive rates and is more conservative than the M8-M8a test [93]. Tests for positive selection can be affected by missing data, indels and alignment quality [95] and the Ensembl data is incomplete in some cases, with missing data randomly distributed across loci. To assess whether this causes a bias in detecting positive selection we compared the distribution of coverage in sites with significant evidence of positive selection under the Bayes Empirical Bayes method [55] to all other sites. In no case was the distribution significantly different (paired t-tests, all loci p > 0.05) suggesting missing data at some sites does not bias the results.

#### Tests for rate shifts & diversifying selection

Branch models allow ω to vary across branches in the phylogeny but not across sites. Branch models can also be used to compare whether or not ω varies between clades or pre-defined lineages. We used this model to compare average ω values across primates to non-primate mammals, and to compare the rates between the three major placental clades, the Euarchontoglires, the Laurasiatheria and Xenarthra + Afrotheria which Meredith *et al*. [92] find to be monophyletic.

A final analysis to detect diversifying selection was performed using Clade model C [34]. Clade models allow a proportion of sites to undergo divergent selection pressures in two or more clades defined *a priori*. These sites may have any value of ω so do not explicitly test for positive selection, or differing amounts of positive selection but may give an indication of differential selection pressures in different clades. Clade model C was compared to the new null model, M2a_rel [96]. Both clade model C and M2a_rel have a proportion of sites evolving under purifying selection and a proportion evolving neutrally in both clades. Where clade model C has a third category where ω varies between the two clades and may be any value >0, M2a_rel has a third category of ω that may be any value >0, but that is shared between clades. Clade model tests were used for the same comparisons as the branch tests, as an independent assessment of shifts in selection.

#### Tests for gene-phenotype co-evolution

Branch models can also be used to compare two phenotypically divergent lineages. We use the branch test here to compare whether several large brained/small brained sister lineages have significantly different *dN/dS* values. A growing number of candidate gene analyses have also sought to explicitly test hypothesised gene-phenotype links by adopting comparative methods to test for an association between *dN/dS* and the phenotype of interest whilst controlling for phylogeny (e.g. [8,97,98]). Here we calculate the root-to-tip *dN/dS* ratio for each species considered and regress these values against brain size using a Phylogenetic Generalised Least Squares model (PGLS), implemented in Bayes Traits ([99] available from http://www.evolution.rdg.ac.uk), to correct for the non-independence of interspecific data caused by their shared evolutionary history. This approach has been used to test for an association between candidate genes, including microcephaly genes, and brain size in primates [8,21,22,66], and between *MCPH1* and *ASPM* and brain size in cetaceans [26,28]. A similar approach has also been adopted in other gene-phenotype studies (e.g. [100,101]). Brain size data were taken from Barton and Capellini [102] and Boddy *et al*. [85].

## Availability of supporting data

The data set supporting the results of this article is included within the article and its additional files.

## Competing interests

The authors declare no competing interests.

## Authors’ contributions

SHM and NIM designed the analyses, SHM collected the data, carried out the analyses and wrote the initial draft of the manuscript. SHM and NIM edited the manuscript.

## Supplementary Material

Additional file 1: Figure S1 Sliding window analysis of STIL **(A)** and WDR62 **(B)**: Ka/Ks = red, Ka = pink, Ks = green. Blue bars indicate regions sequenced from additional anthropoid primates. Click here for file

Additional file 2: Table S1 Accession IDs and phenotypic data. **Table S2.** branch and clade model tests for diversifying selection. **Table S3.** Linking molecular and phenotypic evolution. Click here for file
