# Supplementary material for: Microcephaly genes evolved adaptively throughout the evolution of eutherian mammals
Source: BMC Evol Biol. 2014 Jun 5;14:120. doi: 10.1186/1471-2148-14-120 (PMC4055943; doi:10.1186/1471-2148-14-120)
Supplement: Additional file 1: Figure S1 — Sliding window analysis of STIL (A) and WDR62 (B): Ka/Ks = red, Ka = pink, Ks = green. Blue bars indicate regions sequenced from additional anthropoid primates. [file 1471-2148-14-120-S1.pdf]

A

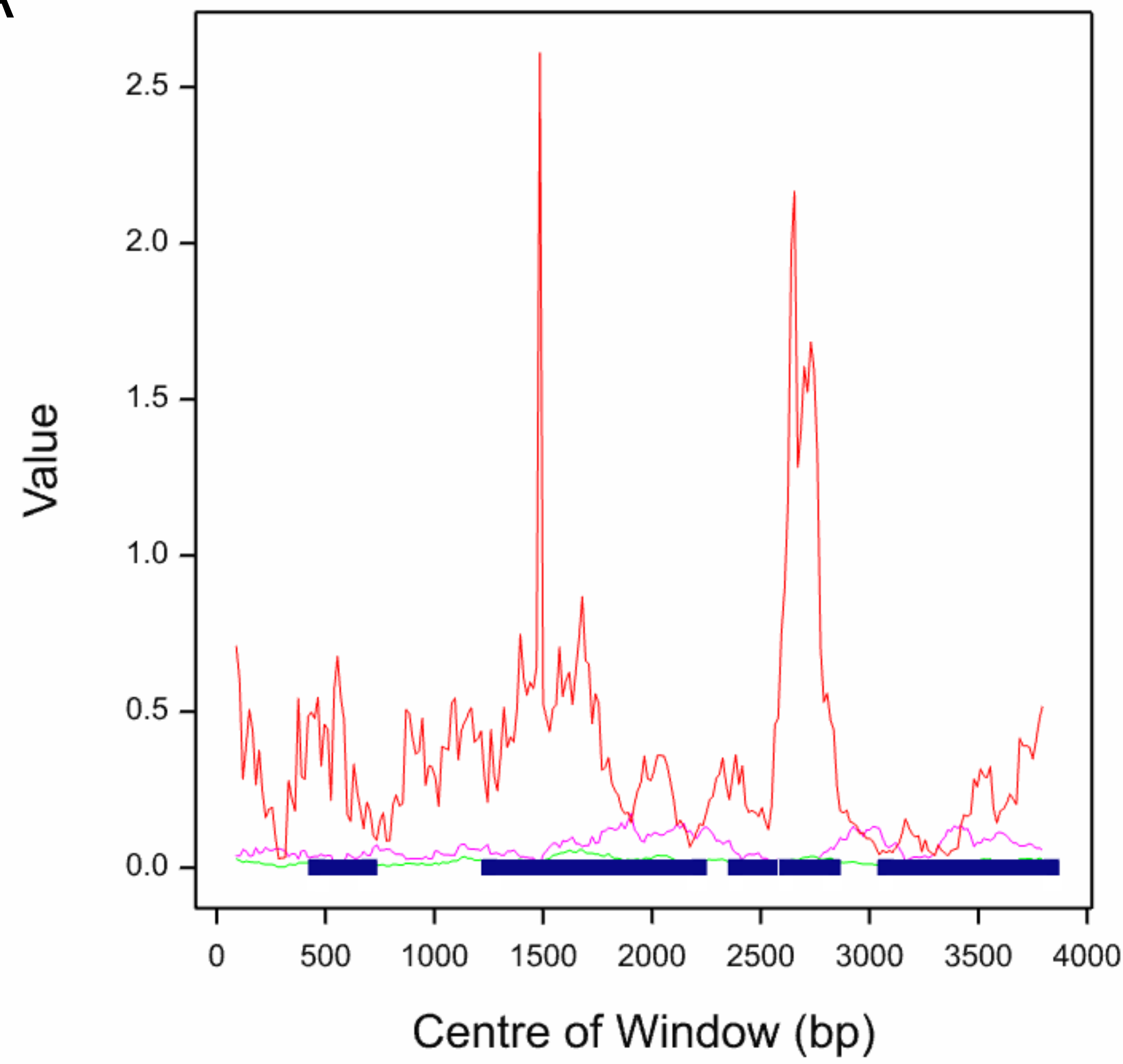

B

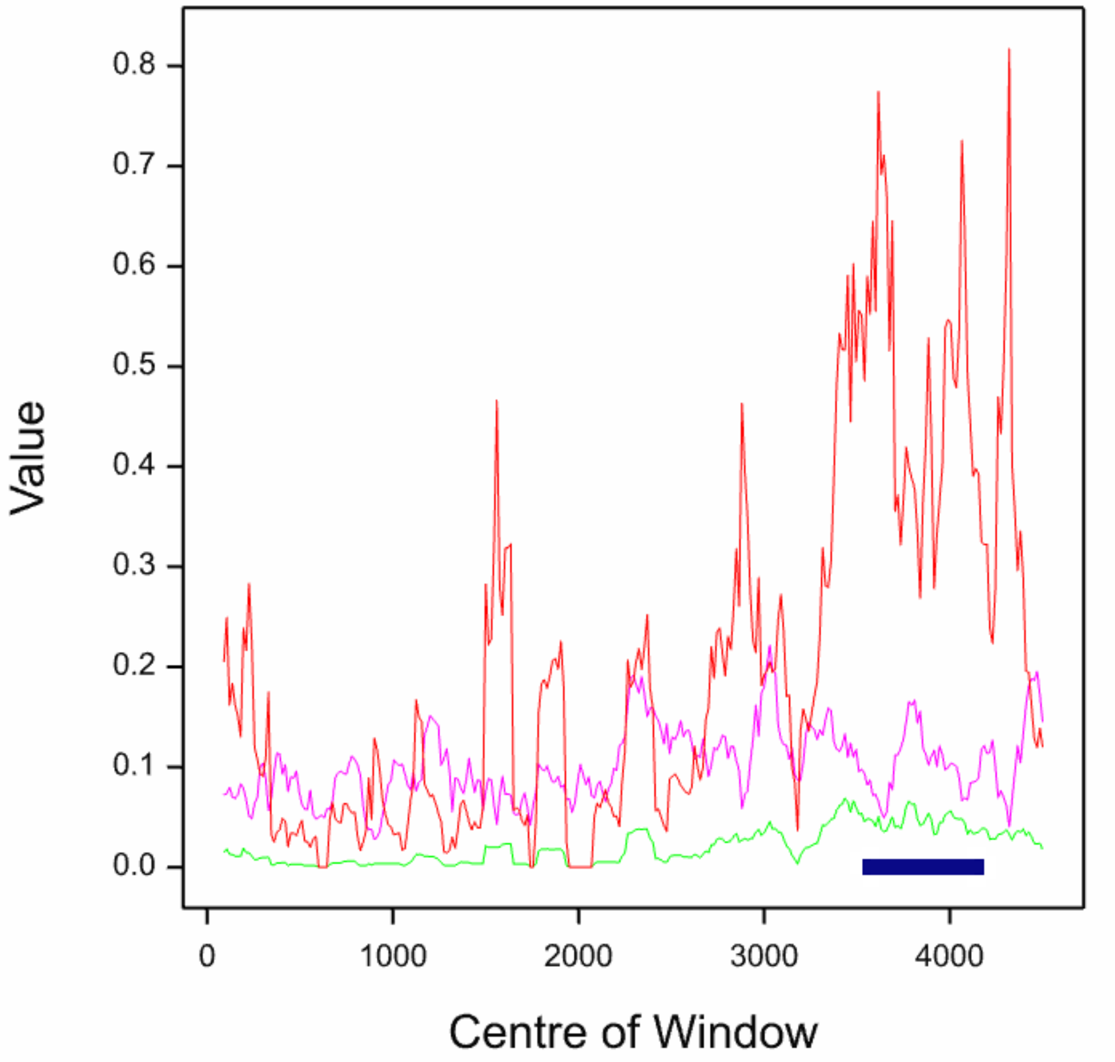

Figure S1:
Sliding window analysis of *STIL* (A) and *WDR62* (B): Ka/Ks = red, Ka = pink, Ks = green. Blue bars indicate regions sequenced from additional anthropoid primates.
